# A simple fluid dynamic model of renal pelvis pressures during ureteroscopic kidney stone treatment

**DOI:** 10.1371/journal.pone.0208209

**Published:** 2018-11-29

**Authors:** Alexandros T. Oratis, John J. Subasic, Natalia Hernandez, James C. Bird, Brian H. Eisner

**Affiliations:** 1 Department of Mechanical Engineering, Boston University, Boston, MA, United States of America; 2 Department of Urology, Massachusetts General Hospital, Harvard Medical School, Boston, MA, United States of America; University of Pennsylvania Perelman School of Medicine, UNITED STATES

## Abstract

Ureteroscopy is an endoscopic kidney stone removal procedure which increases the internal pressure in the renal pelvis, the kidney’s urinary collecting system. Elevated renal pelvic pressure may result in systemic absorption of irrigation fluid and urine, which can increase the risk of postoperative fever and sepsis. Urologists have investigated the effects of various surgical parameters on the renal pelvic pressure. However, it still remains unknown which surgical parameter has the most dominant effect on the renal pelvic pressure over time. Here we develop a physical model that computes the renal pelvic pressure as a function of time based on parameters that can be varied during ureteroscopy. The model is developed by applying pipe network analysis to the regions of the urinary tract that are involved in a representative ureteroscopic procedure. Our model unifies the findings of the previously published studies on this topic; an *ex-vivo* porcine study and an *in-vivo* human study. Furthermore it allows simulation of surgical procedures based on various techniques. Our simulation demonstrates that the two strong regulators of renal pelvis pressure during ureteroscopy are the size of the gap between ureteroscope and ureteral access sheath and the frequency and duration of ureteroscope withdrawal.

## Introduction

Nephrolithiasis is a common disease and affecting approximately one in every ten people in the United States [1]. One of the most common treatments for ureteral and renal stones is ureteroscopic lithotripsy—the use of this treatment modality is increasing and is used for more than 10% of all persons with an acute stone episode. Elevated renal pelvis pressures during ureteroscopic lithotripsy are a risk factor for retrograde flow of urine into the bloodstream via pyelovenous backflow [2–4]. If the urine contains bacteria, this backflow can result in life threatening bacterial infection of the bloodstream [5].

The pressure at which a patient is at risk for pyelovenous backflow and systemic infection is reported to be approximately 40 cm H_2_O and risk is believed to increase with the amount of time that the pressure remains elevated (1 cm H_2_O = 98.1 Pa) [6]. Although high pressures could lead to pyelovenous backflow, some pressure is needed to distend the renal pelvis to better visualize the procedure. Previous clinical studies have examined the effects of various operative parameters and equipment on the renal pelvis pressure [7–9]. The use of ureteral access sheaths (UAS) and low flow rate irrigation have been shown to reduce renal pelvis pressures [10–14], and these results are qualitatively intuitive. Yet, there exists no model that quantitatively encompasses all of these operative parameters, nor is there a sense of if or how this pressure varies with time as a consequence of the renal pelvis compliance. Indeed, only two of these clinical studies mention time, specifically the time-interval over which mean pressures are evaluated [12, 14]. An understanding of how the renal pelvis pressure varies with time based on surgically-relevant parameters is key to estimating the peak and average pressures over the surgery, as well as the role of time-dependent surgical choices, such as the frequency and duration of ureteroscope removal during the procedure.

Here, we develop a model for the time-varying pressure and flow in the urinary system to understand how varying different surgical inputs affects renal pelvis pressures. The model provides insight into how the various parameters affect the characteristic pressures and timescales and quantitatively agrees with the available time-dependent clinical data. Finally, we extend our analysis to evaluate the effect of the ureteroscope withdrawal frequency on the renal pelvis pressure, which by extension can inform surgeons how these choices might affect the risk of pyelovenous backflow and post-operative sepsis.

## Methods

### Model configuration

Unlike past fluid dynamics models of urinary systems that focus on a single conduit, such as the ureter [15] or the urethra [16], we find it most natural to model the ureteroscopic procedure as flow through a series of distinct connected pipes. This pipe network analysis requires an understanding of the geometry of the system being modeled, and specifically how it can be parsed into connected pipes.

**Fig 1:**
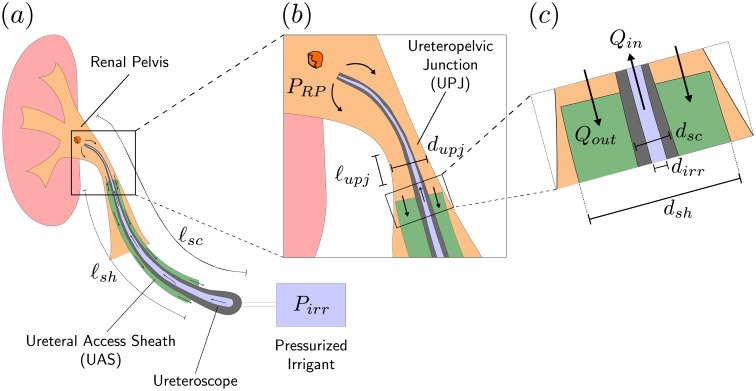
Schematic of the regions of fluid flow during ureteroscopy and their relevant parameters. **(a)** The pressure of the irrigant *P*_*irr*_ develops a flow through the ureteroscope with length *ℓ*_*sc*_ into the renal pelvis. The schematic includes a ureteral access sheath (UAS) with length *ℓ*_*sh*_ through which the irrigant exits the renal pelvis. **(b)** Magnified view of the ureteropelvic junction (UPJ) defined by its diameter *d*_*upj*_ through which the ureteroscope passes from the end of the UAS into the renal pelvis. The UAS is often placed a short distance *ℓ*_*upj*_ distal to the UPJ. **(c)** Magnified view of the end of the UAS. Liquid enters the renal pelvis with flowrate *Q*_*in*_ through the irrigation channel with diameter *d*_*irr*_ and exits with flowrate *Q*_*out*_ through the annular region between the UAS and the ureteroscope defined by their diameters *d*_*sh*_ and *d*_*sc*_ respectively.

In a typical ureteroscopy procedure, a ureteroscope with length *ℓ*_*sc*_ is either inserted into the renal pelvis alone or it is inserted through a ureteral access sheath (UAS) with length *ℓ*_*sh*_. The ureteroscope consists of an irrigation channel with diameter *d*_*irr*_ that is connected to a pressurized irrigant, usually saline. Due to the elevated pressure of the irrigant *P*_*irr*_, a flow develops and drives the irrigant through the irrigation channel of the ureteroscope into the renal pelvis (Fig 1a). Often, the UAS is positioned a short distance *ℓ*_*upj*_ distal to the ureteropelvic junction (UPJ), which serves as a functional valve between the renal pelvis and proximal ureter. Once saline reaches the renal pelvis it outflows through the region between the UPJ and the ureteroscope and subsequently through the region between the ureteroscope and the UAS, if a sheath is present, or between the ureteroscope and the ureter (Fig 1a and 1b).

As saline flows out of the ureteroscope, some of the liquid distends the renal pelvis by a volume Δ*V* while the rest of the liquid flows out through the UAS or adjacent to the ureteroscope. Because the renal pelvis is elastic, the change in volume Δ*V* can be related to a change in pressure through a linear constitutive relation Δ*P* = *K*Δ*V* where *K* is the renal pelvis stiffness. This stiffness itself can vary with pressure, volume and time, as would occur if the tissue were modeled as a Neohookean solid or a viscoelastic material [17]. In the current study, we assume a constant stiffness, whose value we choose as *K* = 0.4 ± 0.3 cm H_2_O/mL (1 cm H_2_O/mL = 9.8 ×10^-5^ Pa/m^3^). This value is determined from the eight different renal pelvis pressure-volume relationships reported by Mortensen *et al*. [18]. The final value is computed as the mean and the associated error is calculated through a t-test.

As the liquid (e.g. saline irrigation fluid and/or urine) is nearly incompressible, conservation of mass requires that the volume of liquid retained in the renal pelvis Δ*V* be related to the difference in the inflow rate *Q*_*in*_ and outflow rate *Q*_*out*_ (Fig 1c). Symbolically this mass conservation can be expressed in terms of the change in volume as $\Delta V=\int_{0}^{t}\;\left(Q_{in}-Q_{out}\right)dt$, where *t* is time. Taking the time derivative of the mass conservation expression and combining it with the constitutive relationship results in a differential equation for the renal pelvis pressure *P*_*RP*_ as a function of time *dP*_*RP*_/*dt* = *K*(*Q*_*in*_ − *Q*_*out*_).

The rates of inflow *Q*_*in*_ and outflow *Q*_*out*_ are calculated using Poiseuille’s law $\Delta P_{i}=R_{i}Q_{i}$. Here, *i* is an index for either the inflow or outflow, Δ*P*_*i*_ is the respective pressure difference, and $R_{i}$ is the hydraulic resistance. Poiseuille’s law is a simplified form of the conservation of momentum and is appropriate because the flow can be modeled as Newtonian, incompressible, laminar, and quasi-static with no-slip boundary conditions for the velocity at the solid walls. The hydraulic resistance combines both viscous and geometric effects. For example, for a cylindrical tube, the hydraulic resistance is $R_{i}=128\mu \ell_{i}/\pi d_{i}^{4}$, where *ℓ*_*i*_ and *d*_*i*_ are the length and diameter of the tube respectively and *μ* is the fluid viscosity, approximately 1 cP for saline (1 cP = 10^-3^ Pa⋅s).

### Governing equation

Applying Poiseuille’s law to each region of saline flow formulates the governing equation of the renal pelvis pressure:

$$\begin{array}{c} \frac{dP_{RP}}{dt}+\frac{K}{R_{in}}(1+\frac{R_{in}}{R_{out}})P_{RP}=\frac{K}{R_{in}}P_{irr}, \end{array}$$ (1)

where $R_{in}$ and $R_{out}$ are the inflow and outflow resistances respectively defined as:

$$\begin{array}{c} R_{in}=\frac{128\mu }{\pi }\frac{\ell_{sc}}{d_{irr}^{4}} \end{array}$$ (2a)

$$\begin{array}{c} R_{out}=\frac{128\mu }{\pi }(\frac{\ell_{sh}}{{\tilde{d}}_{sh}^{4}}+\frac{\ell_{upj}}{{\tilde{d}}_{upj}^{4}}). \end{array}$$ (2b)

Since the outflow resistance $R_{out}$ consists of annular regions, the respective diameters are computed as:

$$\begin{array}{c} {\tilde{d}}_{sh}^{4}=d_{sh}^{4}-d_{sc}^{4}-\frac{{\left(d_{sh}^{2}-d_{sc}^{2}\right)}^{2}}{\text{ln}(d_{sh}/d_{sc})} \end{array}$$ (3a)

$$\begin{array}{c} {\tilde{d}}_{upj}^{4}=d_{upj}^{4}-d_{sc}^{4}-\frac{{\left(d_{upj}^{2}-d_{sc}^{2}\right)}^{2}}{\text{ln}(d_{upj}/d_{sc})}. \end{array}$$ (3b)

We use the same theoretical approach to model the fluid outflow and decay of renal pelvis pressure when the ureteroscope is withdrawn. When the ureteroscope is withdrawn (as would occur when during basket extraction of stone fragments), there is no longer flow into the renal pelvis so that the conservation of mass includes only the outflow rate. Under this condition, the governing equation is modified to:

$$\begin{array}{c} \frac{dP_{RP}}{dt}+\frac{K}{R_{w}}P_{RP}=0, \end{array}$$ (4)

where $R_{w}$ is the withdrawal resistance consisting of the same UPJ and UAS regions but with the scope withdrawn, these regions become circular from annular:

$$\begin{array}{c} R_{w}=\frac{128\mu }{\pi }(\frac{\ell_{upj}}{d_{upj}^{4}}+\frac{\ell_{sh}}{d_{sh}^{4}}). \end{array}$$ (5)

The two governing Eqs (1) and (4) require their own initial conditions. For Eq (1), the initial pressure is *P*_*RP*_(*t* = 0) = *P*_0_; whereas for the case of ureteroscope removal, the initial condition of Eq (4) is *P*_*RP*_(*t* = *t*_*r*_) = *P*_*r*_, where *t*_*r*_ is the time at which the ureteroscope is removed and *P*_*r*_ is the instantaneous pressure at that time. Solving these equations analytically indicates that when the ureteroscope is inserted, the renal pelvis pressure rises with time and follows an inverse exponential decay:

$$\begin{array}{c} P_{RP}=P_{irr}{\left(1+\frac{R_{in}}{R_{out}}\right)}^{-1}\left(1-e^{-t/\tau_{in}}\right)+P_{0}e^{-t/\tau_{in}} \end{array}$$ (6a)

$$\begin{array}{c} \tau_{in}=\frac{R_{in}}{K}{\left(1+\frac{R_{in}}{R_{out}}\right)}^{-1}, \end{array}$$ (6b)

where *τ*_*in*_ is the characteristic rise. Whereas, when the ureteroscope is removed, the renal pelvis decays exponentially with time:

$$\begin{array}{c} P_{RP}=P_{r}e^{-\left(t-t_{r}\right)/\tau_{out}} \end{array}$$ (7a)

$$\begin{array}{c} \tau_{out}=\frac{128\mu }{\pi K}(\frac{\ell_{upj}}{d_{upj}^{4}}+\frac{\ell_{sh}}{d_{sh}^{4}}). \end{array}$$ (7b)

The time and pressure start from when the ureteroscope is removed at an initial pressure of *P*_*r*_ and decay exponentially to a gauge pressure of zero (baseline) with a characteristic time *τ*_*out*_.

### Representative values for model parameters

The values of the geometric parameters used throughout the model are summarized in Table 1. When modeling procedures without a UAS, the sheath length *ℓ*_*sh*_ and diameter *d*_*sh*_ are replaced with the ureter length *ℓ*_*ur*_ and ureter diameter *d*_*ur*_ respectively. The length of the ureter *ℓ*_*ur*_ is approximately 25 cm [19]. A precise value for the ureter diameter *d*_*ur*_, is less clear as extreme values can range between 5.7—15.9 Fr (3 Fr = 10^-3^ m) [20].

**Table 1:** Representative parameter values used in the model. The values selected for the ureteroscope correspond to the Karl Storz Flex-X2. Note that the ureter and UPJ diameters are the relaxed sizes and are adjusted, as described in the text, when distended by the ureteroscope. *Note that the diameter used is the tip diameter of the Karl-Storz Flex X2 ureteroscope, which is a reasonable approximation for the entire diameter of the shaft.

Irrigation Pressure	*P*_*irr*_	150, 200 cm H_2_O
**Ureteroscope**		
Diameter*	*d*_*sc*_	7.5 Fr
Working Channel Diameter	*d*_*irr*_	3.6 Fr
Working Length	*ℓ*_*sc*_	67 cm
**Ureteral Access Sheath**		
Diameter	*d*_*sh*_	10/12, 12/14, 14/16 F
Length	*ℓ*_*sh*_	35 cm
**Physiological Parameters**		
UPJ Diameter	*d*_*upj*_	1.3 Fr
UPJ Length	*ℓ*_*upj*_	0.5 cm
Ureter Diameter	*d*_*ur*_	8.5 Fr
Ureter Length	*ℓ*_*ur*_	25 cm
Renal pelvis Stiffness	*K*	0.41±0.3 cm H_2_O/mL
Fluid Viscosity	*μ*	1 cP

The diameters of the ureter, *d*_*ur*_, and UPJ, *d*_*upj*_, are generally larger than the ureteroscope tip diameter *d*_*sc*_ during ureteroscope insertion. Therefore, it is simplest to estimate the effective, stretched diameters relative ureterscope diameter. For the model presented here, the stretched ureter and UPJ diameters were approximated to be 14% and 11% larger than the diameter of the ureteroscope respectively, as these values provided results that were consistent with the published data in the prior study by Rehman *et al*. [12].

Under conditions in which the ureteroscope was not present, such as during basket extraction of stone fragments, the non-dilated, nominal diameters for the UPJ and ureter were used (Table 1). The nominal diameter of the UPJ was estimated by combining Poiseuille’s law with the Whitaker test, a urodynamic test that combines flow with pressure difference between the renal pelvis and bladder and is used to assess upper urinary tract obstruction [21]. Although the Whitaker test is considered outdated by urological standards, it is still satisfactory to get an estimate for the UPJ diameter. For an unobstructed ureter, the pressure difference across ureter is approximately Δ*P* = 15 cm H_2_O for a flow rate of approximately *Q* = 15 mL/s [22, 23]. The hydraulic resistance was computed with these values and results in an effective UPJ diameter of *d*_*upj*_ = 1.3 Fr.

### Model validation and extension to ureteroscope withdrawal

To test the validity of our model, the conditions of two previously published studies [12, 14] were replicated so that the model results could be directly compared to these clinical data sets. Rehman *et al*. used the Storz Flex-X2 (Karl Storz, Tuttlingen Germany) in a pig model and reported mean renal pelvis pressures after three minutes [12]; whereas, Shao *et al*. used the Wolf 8/9.8 Fr semirigid ureteroscope (Richard Wolf, Vernon Hills, IL) in a human study and reported mean pressures after five minutes [14]. The Rehman *et al*. study treated kidney stones in the renal pelvis; in contrast, the Shao *et al*. study treated kidney stones in various locations of the ureter. As such, our model was modified to account for the flow differences that occur when treating a kidney stone that is located in the ureter. Specifically, a hydraulic resistance is included in addition to the UPJ, that models the flow through the ureter whose length depends on the kidney stone location. The dimensions of the ureteroscope and UAS, as well as irrigation pressures, used in those studies were input into our model and the corresponding theoretical time dependent pressure was computed. We then compute a running time-averaged pressure, defined as ${\bar{P}}_{RP}\;=\;\frac{1}{t}\int_{0}^{t}\;P_{RP}\left(\tilde{t}\right)d\tilde{t}$.

Once validated, we use our model to simulate the pressure resulting from a ureteroscopic lithotripsy procedure with four different withdrawal options. For each simulated procedure, the irrigation pressure is 150 cm H_2_O and the total time spent with the ureteroscope in the renal pelvis was 20 minutes (Table 2). In the first case the procedure is carried out without removing the ureteroscope for a total of 20 minutes. For the remaining three scenarios, the ureteroscope is repeatedly removed and reinserted at set intervals so that the total insertion time is still 20 minutes. The withdrawal time is always smaller in duration than the insertion time and the number of withdrawals is always one less than insertion. These four model scenarios were repeated for three different UAS sizes: 10/12 Fr, 11/13 Fr and 12/14 Fr. To evaluate the effect of the withdrawal on the renal pelvis pressure, we report the relative time-averaged pressure decrease for the three options in which the ureteroscope is withdrawn. The relative pressure is with respect to the case in which the ureteroscope is not withdrawn.

**Table 2:** The four scenarios used to evaluate how ureteroscope removal affects the renal pelvis pressure. Each option has the same cumulative insertion time but different cumulative withdrawal time. The total procedure times is thus different for the four different options. The model procedures are repeated for a 10/12, 11/13 and 12/14 Fr UAS, as well as no UAS.

Option #	Insertion	Withdrawal	Total Procedure
1	20 min × 1	0 min	20 min
2	10 min × 2	5 min × 1	25 min
3	4 min × 5	2 min × 4	28 min
4	2 min × 10	1 min × 9	29 min

## Results and discussion

### Model solution

**Fig 2:**
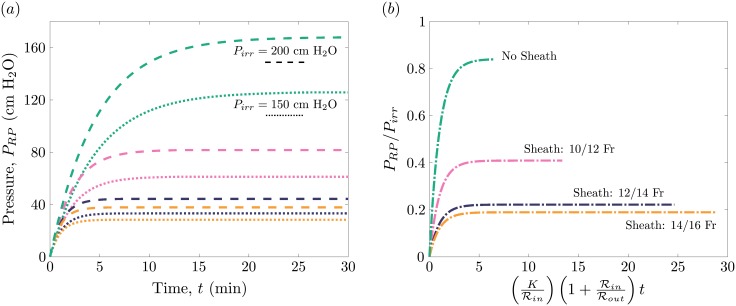
Theoretical prediction of renal pelvis pressure as a function of time for varying sheath sizes and irrigation pressures. (*a*) Our model’s theoretical prediction of the renal pelvis pressure in cm H_2_O over time. The dashed and dotted lines correspond to irrigation pressures of *P*_*irr*_ = 200 cm H_2_O and *P*_*irr*_ = 100 cm H_2_O respectively. The sheath sizes considered here are *r*_*sh*_ = 10/12 Fr, 12/14 Fr and 14/16 Fr as well as a case without the sheath. (*b*) These eight curves collapse onto four distinct curves when plotting the pressure relative to the irrigation pressure and the time with respect to the characteristic time.

Inputting the representative values (Table 1) into our model solution (Eq (6)) leads to the pressure curves illustrated in Fig 2. The different colors corresponds to four different sheath choices; no sheath, 10/12 Fr, 12/14 Fr and 14/16 Fr. Simultaneously, the dotted and dashed line styles correspond to an irrigation pressure of 150 cm H_2_O and 200 cm H_2_O respectively. Note that this maximum pressure increases for larger irrigation pressures (Fig 2a). In addition, the maximum pressure drops when the sheath is increased due to the larger cross-sectional area the fluid can escape through between the sheath and the ureteroscope. Hence, we find that our theoretical description qualitatively captures the effects of the irrigation pressure and sheath size on the renal pelvis pressure [7, 10–14].

Quantitatively, the model provides falsifiable predictions as to how the pressure rises with time and the maximum pressure it reaches. In all cases, the pressure rises over a duration of several minutes, at which point the two larger sheaths plateau near or below 40 cm H_2_O, the value considered to be the threshold for pyelovenous backflow. In contrast, the pressure when using a 10/12 Fr sheath or when using no sheath at all continues to rise for several more minutes and, for these particular irrigation pressures, plateaus at values exceeding 40 cm H_2_O. Note that the maximum pressure in all cases is below the irrigation pressure. Indeed, for a given sheath option the model predicts the renal pelvis pressure will plateau at a pressure that is a set fraction of the irrigation pressure (Fig 2b). In the absence of a sheath the maximum pressure is approximately 80% of the irrigation pressure; whereas, for the 10/12 Fr sheath the maximum pressure is approximately 40%, and for 12/14 Fr and 14/16 Fr sheaths the maximum pressure is approximately 20% of the irrigation pressure. Eq (6) also presents a characteristic time *τ*_*in*_, based on the geometry of compliance of the system. In Fig 2b, when all of the eight curves in Fig 2a are rescaled by the irrigation pressure and this characteristic time, they collapse onto curves based solely on the sheath option.

A key factor influencing both the maximum pressure and the rise time is the gap between the ureteroscope and the sheath and the gap between the ureteroscope and the UPJ. Because the outflow resistance is inversely proportional to the gap size, $R_{out}∼{\tilde{d}}^{-4}$, a slight increase in the gap would decrease both the maximum pressure and the associated time. Indeed, increasing the gap size by 1 Fr, by moving from a “no sheath” option to a 10/12 Fr sheath, reduces the maximum pressure by a half. Similarly, doubling the gap size to 4.5 Fr by using the 12/14 Fr sheath, reduces the maximum pressure again by half. Yet when the gap size is increased by another 2 Fr by moving to the 14/16 Fr sheath, the change in maximum pressure is not as dramatic. Note that the outflow resistance depends on both UPJ and sheath gap sizes. For small sheath gap sizes, the influence of the UPJ is negligible and the sheath geometry essentially sets the maximum pressure and the characteristic time. For sheath size larger than 12/14 Fr, the UPJ has an increasingly important role and controls the maximum pressure and the characteristic time.

The renal pelvis stiffness and liquid viscosity also influence the characteristic time. Although the maximum pressure is unaffected by the renal pelvis stiffness, it influences the time it takes to reach that value. The viscosity of the saline and urine is well characterized and essentially constant throughout the procedure. In contrast, uncertainty related to the renal pelvis stiffness is significantly larger and potentially the largest source of error in our model.

### Comparison with reported data

**Fig 3:**
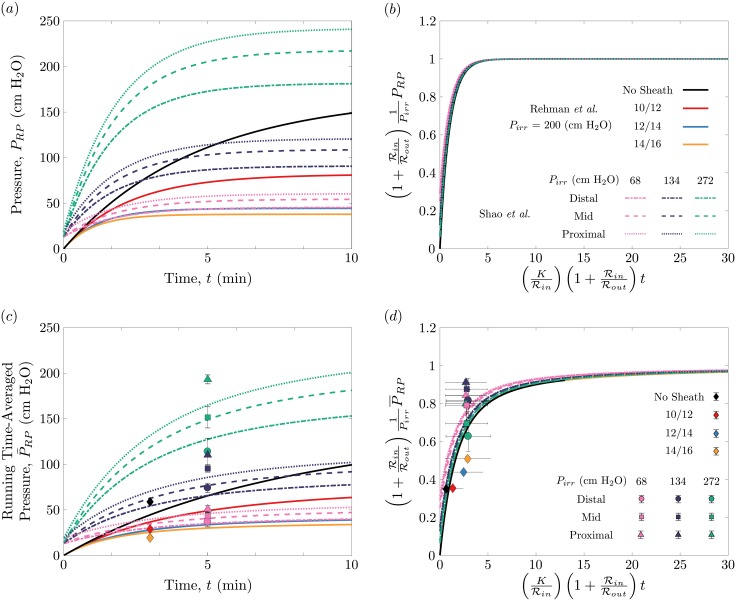
Our predictions for the pressure-time profiles corresponding to the cases reported in Rehman *et al*. [12] and Shao *et al*. [14]. (**a**) Theoretical pressure curves against time for all 13 cases. The solid lines correspond to the different sheath sizes studied by Rehman *et al*. in which the irrigation pressure is *P*_*irr*_ = 200 cm H_2_O. The remaining curves are the cases studied by Shao *et al*. in which the pressure in the renal pelvis is examined for stones located at three different locations in the ureter for three different irrigation pressures. (**b**) These 13 pressure curves nearly collapse when the renal pelvis pressure is normalized by the maximum pressure and time is normalized by the characteristic rise time. (**c**) The running time-averaged pressure of these curves can be related to past clinical data sets. The diamonds correspond to the data reported by Rehman *et al*. while the circles, squares and triangles denote the data reported by Shao *et al*. (**d**) These average pressure curves collapse when normalized by the maximum pressure and plotted against normalized time.

We proceed by taking our theoretical results and comparing them to the data reported in the studies by Shao *et al*. and Rehman *et al*. We replace the representative values we reported in Table 1 with the values that each of these studies used in their clinical studies, such as the ureteroscope dimensions, irrigation pressures and UAS sizes. Fig 2a reports how the pressure in each of case of each study varies with time. The solid curves correspond to the Rehman *et al*. cases, in which the irrigation pressure was kept constant but the UAS size was varied. The dotted, dashed and dot-dashed curves correspond to the decreasing irrigation pressures reported in the Shao *et al*. cases, with the different colors indicating a different kindey stone location along the ureter. Because baseline pressures were reported in the Shao *et al*. study, the pressure curves do not start from zero pressure. Note that the trends of these curves are identical to the ones we showed in Fig 2a, which should be expected as Eq (6a) continues to be used and only the parameter values have been changed. Fig 3b nearly collapses all 13 curves in Fig 3a by rescaling time with the rise time and pressure with the maximum pressure. This dimensionless plot highlights what percentage of the maximum pressure is reached at a particular time relative to the rise time. The purpose of normalizing the dimensional data—a common practice in fluid mechanics—is to illustrate how particular groupings of parameters can collapse (or reduce the dimensionality of the data). Here the pressure–time profiles in Fig 3a, which vary in terms of the parameter values, follow the same behavior, and nearly collapse onto a single curve when appropriately non-dimensionalized. The variation in curves in Fig 2b can be attributed to the differences in baseline pressure and stone position in the Shao *et al*., and because these variations are small, it is clear that they do not have a leading-order effect on these pressure-time profiles.

Because the pressures reported in the studies by Shao *et al*. and Rehman *et al*. are not instantaneous but rather time-averaged, it is helpful for comparison to calculate a running time-averaged pressure. Integrating Eq (6a) and dividing by the elapsed time, we find that the time-averaged renal pelvis pressure follows:

$$\begin{array}{c} {\bar{P}}_{RP}=P_{irr}{\left(1+\frac{R_{in}}{R_{out}}\right)}^{-1}+\frac{\tau_{in}}{t}[P_{irr}{\left(1+\frac{R_{in}}{R_{out}}\right)}^{-1}-P_{0}]\left(1-e^{-t/\tau_{in}}\right). \end{array}$$ (8)

Fig 3c compares the analytical time-averaged pressure with the data published in the prior studies by Rehman *et al*. (diamond points) and Shao *et al*. (circle, square and triangle points). The running time-averaged pressure curves indicate the mean pressure over an elaspsed time *t*. These curves also collapse when rescaled by the maximum pressure and plotted against a dimensionless time (Fig 3d). Again, the slight differences are due to the initial pressure *P*_0_, and the additional hydraulic resistances that arise for the kidney stones located in the ureter. Yet, the fact these differences are small indicates that these parameters do not have a dominant effect on the overall pressure profiles. The clinical data sets are plotted in both Fig 3c and 3d and are consistent with our theoretical predictions of Eq (8). Even though our model lacks precision, we are encouraged to see the consistency between the theoretical prediction and the clinical data sets, especially considering the uncertainty in the renal pelvis compliance, and this consistency provides some assurance to our modeling approach.

### Extension to withdrawal strategies

**Fig 4:**
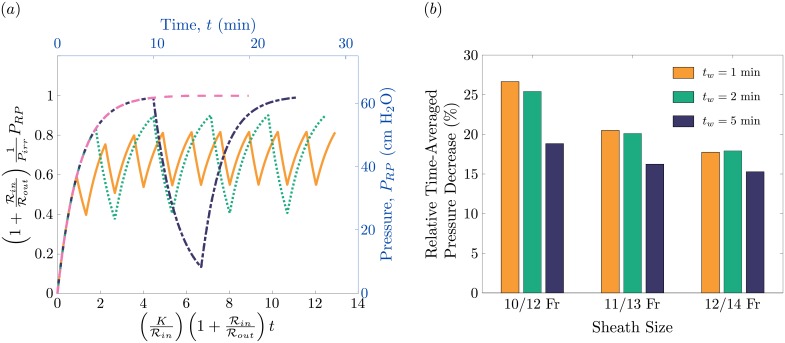
Renal pelvis pressure for the various withdrawal strategies presented in Table 2. (*a*) The renal pelvis against time for a 10/12 Fr sheath. The dotted line corresponds to Option #1, the dot-dashed line to Option #2, the dotted line to Option #3 and the solid line to Option #4. Note that the left and bottom axes present the pressure and time in dimensionless terms respectively, while the right and top axes present the pressure and time in dimensional values. (*b*) Relative time-averaged pressure decrease for the different options against the three different sheath sizes 10/12 Fr, 11/13 Fr and 12/14 Fr. The relative decrease is with respect to the time-averaged pressure the “no withdrawal” option (Option #1). Note that *τ*_*w*_ stands for withdrawal time.

We extend our analysis by evaluating how the frequency of the ureteroscope withdrawal during the procedure affects the renal pelvis pressure. The pressure profiles for the four different options of Table 2 using a UAS size of 10/12 Fr are illustrated in Fig 4a. Note that curves are plotted in both dimensional (top and right axes) and dimensionless (bottom and left axes) forms. Even though the total procedure time varies between each option, the time spent with the ureteroscope inserted in the renal pelvis remains constant. The dashed curve—corresponding to the no-withdrawal option—ends at exactly 20 minutes, while the dot-dashed, dotted and solid curves correspond to 5, 2 and 1 minute regular withdrawal intervals respectively. The no-withdrawal option and the 5 minute withdrawal option reach the maximum pressure, yet result in shorter total operative times. By contrast, the 2 and 1 minute withdrawal options result in longer operative times, but do not reach their potential peak pressures. Since the time-averaged pressure of the no-withdrawal is always larger than the remaining options, we can assess the effect of ureteroscope withdrawal by computing the relative time-averaged pressure of each withdrawal option with respect to the no-withdrawal case.

Fig 4b reports the relative time-averaged pressures for the three withdrawal options not only for the 10/12 Fr sheath but also for a 11/13 Fr sheath and 12/14 Fr sheath. The maximum pressure decrease, approximately 25%, occurs for the 10/12 Fr sheath when the withdrawal intervals are the shortest. Note that even though the pressure decrease may be largest in these cases, the total operative time is also increased. As the sheath size is increased, the difference in relative pressure between the different withdrawal options decreases. It might be initially surprising that, for the 12/14 Fr sheath, the pressure decrease at 2-minute withdrawals is slightly larger than for either the 1-minute or 5-minute withdrawals. This non-monotonic behavior can be rationalized by considering both the difference in operative times and peak pressures reached. Fig 4b also reveals that the effect of varying withdrawal duration and frequency on the time-averaged pressure diminishes with increasing sheath size. These effects suggest, that when treating a patient with a history of infection, the extra operative time might be worth taking if the pressure is to remain at the lowest possible levels throughout the procedure. By contrast, when a patient without a history of infection is treated, a single but longer withdrawal option or a no-withdrawal option may be preferred, as it results in a shorter operative time.

To give our model potential clinical relevance, we have constructed it to be flexible enough to easily include various techniques and equipment that the surgeon can vary. One way surgeons can alter their techniques to positively affect renal pelvis pressure is to control the surgical parameters, such as the sheath size, ureteroscope size, and irrigation pressure. Another parameter that a surgeon could *potentially* vary is the frequency and duration of ureteroscope withdrawal. In current practice, many surgeons complete lasering, and then repeatedly basket fragments (i.e. withdraw the ureteroscope) and replace it at very short intervals, typically less than 30 seconds. However, our modeling, which is based on novel intervals and duration of ureteroscope withdrawal, suggests that there may be advantages to consider different withdrawal strategies in an effort to minimize renal pelvis pressures.

## Conclusion

It has long been postulated that urosepsis after ureteroscopy occurs due to pyelovenous backflow of bacteria in the urine or harbored within a stone that is fragmented. Pyelovenous backflow was first described in 1856 by Gigon while evaluating kidney specimens and was further explored in the 1920s by Hinman, Lee-Brown, and Laidley, who noted the phenomenon of pyelovenous backflow across animal species (sheep, ox, calf, living rabbits) and *ex-vivo* human kidneys [2–4]. Decreasing renal pelvis pressure to lower the risk of sepsis is one reason that there has been a renewed interest in the use of UAS in ureteroscopy over the past 15 years, largely influenced by the work of Kourambas *et al*. in 2001 [13, 24]. This association between renal pelvis pressure, sepsis risk, and UAS usage is further corroborated by a recent multi-center trial of ureteroscopy, which demonstrated that the use of UAS decreased post-ureteroscopy sepsis rates by 50% [5]. In the current study, we set out to create a model that evaluates renal pelvis pressures and with the goal of identifying parameters that could be modified in order to prevent urosepsis. We demonstrated that our model was consistent with the experimental results of previously published studies and could also be extended to simulate procedures of varying techniques. For instance, when considering periodic ureteroscope withdrawals, the model predicts a trade off between renal pelvis pressure and operative time. Ureteroscope withdrawal decreases the renal pelvis pressure at the expense of a longer total operative time and the magnitude of this effect is inversely related to the UAS diameter.

We believe that there are two important future directions for this research. One would be to better understand and reduce the uncertainty of our model. Mathematical modeling has known limitations. We were encouraged to see that our model is consistent with the outcomes of previously published studies by Rehman and Shao [12, 14], but to bolster confidence of this model as a useful tool in endourology, additional *in-vivo* animal and human studies are necessary, especially related to the compliance of the renal pelvis. It is also noteworthy that for the case of “no sheath”, we did not account for any bladder outlet resistance or bladder filling. These parameters are less relevant when using a ureteral access sheath, as the sheath bypasses the bladder and outlet to drain urine directly out of the patient. A second direction would be to use model described above to create a useful tool for the endourologist. To accomplish this objective, we envision a web-based or perhaps mobile device application that could inform the urologist’s technique on a case by case basis. For instance, we selected the Karl-Storz ureteroscope for the simulated ureteroscopy because we needed a benchmark. However, the power of the model is that one can vary the inputs to various manufacturer specifications. Future studies could compare how various ureteroscopes perform given a particular sheath size.

This manuscript is not to argue for or against the use of techniques which lower renal pelvis pressures (such as use of UAS)—rather, it is to help the surgeon tailor techniques to each individual patient in order to reduce post-operative infectious complications. These patient-specific decisions can include making choices about irrigation technique, ureteroscope size, use of UAS, UAS diameter, and basketing technique. Our findings will certainly have greater relevance for patients who are at risk for infectious complications, as opposed to those who are not—i.e. patients with prior episodes of infection, stones colonized with bacteria, or struvite stones. Our model will allow the surgeon to input the parameters of their proposed technique (i.e. use of UAS, UAS size, ureteroscope diameter, irrigation rate) and then to evaluate the relative effects of varying those parameters on renal pelvis pressures for the procedure. It is our hope that with further refinement using clinical data, models such as ours can help surgeons better understand the relationship between their equipment choices, surgical techniques, and relative renal pelvis pressures.
